# Epigenetic Regulation of Chondrocytes and Subchondral Bone in Osteoarthritis

**DOI:** 10.3390/life12040582

**Published:** 2022-04-14

**Authors:** Hope C. Ball, Andrew L. Alejo, Trinity Kronk, Amanda M. Alejo, Fayez F. Safadi

**Affiliations:** 1 Department of Anatomy and Neurobiology, Northeast Ohio Medical University, Rootstown, OH 44272, USA; aalejo@neomed.edu (A.L.A.); tsamson@neomed.edu (T.K.); amalejo@neomed.edu (A.M.A.); 2 Musculoskeletal Research Group, Northeast Ohio Medical University, Rootstown, OH 44272, USA; 3 GPN Therapeutics, Inc., REDI Zone, Rootstown, OH 44272, USA; 4 Department of Orthopaedic Surgery, Akron Children’s Hospital, Akron, OH 44308, USA; 5 Rebecca D. Considine Research Institute, Akron Children’s Hospital, Akron, OH 44302, USA

**Keywords:** osteoarthritis, epigenetics, methylation, bone, cartilage

## Abstract

The aim of this review is to provide an updated review of the epigenetic factors involved in the onset and development of osteoarthritis (OA). OA is a prevalent degenerative joint disease characterized by chronic inflammation, ectopic bone formation within the joint, and physical and proteolytic cartilage degradation which result in chronic pain and loss of mobility. At present, no disease-modifying therapeutics exist for the prevention or treatment of the disease. Research has identified several OA risk factors including mechanical stressors, physical activity, obesity, traumatic joint injury, genetic predisposition, and age. Recently, there has been increased interest in identifying epigenetic factors involved in the pathogenesis of OA. In this review, we detail several of these epigenetic modifications with known functions in the onset and progression of the disease. We also review current therapeutics targeting aberrant epigenetic regulation as potential options for preventive or therapeutic treatment.

## 1. Introduction

Epigenetics, as a word, has been present in the biological vocabulary since it was coined by C.H. Waddington in the 1930s [1]. Since then, the concepts this word describes have undergone extensive changes, from a broad overall postulation on the workings of molecular development to the more modern term used to refer to a multitude of genetic and transcriptomic chromatic regulators [1,2]. The scope and breadth of these regulators can vary depending on context but commonly incorporate nucleotide or amino acid modifications such as methylation/demethylation, acetylation/deacetylation, phosphorylation, glycosylation, ubiquitination, transposable elements, non-coding RNAs (ncRNAs), small-interfering RNAs (siRNAs), and microRNAs (miRNAs). More recently, scientists have been expanding studies of these sporadic or temporal cellular events to determine the influence of environmental changes during development (environmental epigenetics) and/or inherited (transgenerational epigenetics) on epigenetic regulators [3,4]. Furthermore, studies are beginning to shed light on how epigenetic changes affect normal physiological functions.

Epigenetic modifications during aging have been shown to negatively affect tissue physiological function by themselves or in conjunction with various environmental factors, such as weight, stress, or drug/alcohol usage [5,6]. Recent studies have linked changes in epigenetic regulation to the development and/or progression of a wide range of human diseases. DNA demethylation, aberrant histone methylation, and histone enzymatic activity have been linked to cancer and tumor metastases [7,8,9]. Similarly, epigenetic dysregulation has been linked to cardiovascular disease [5,10], and diabetic kidney disease [11,12]. 

**Figure 1:**
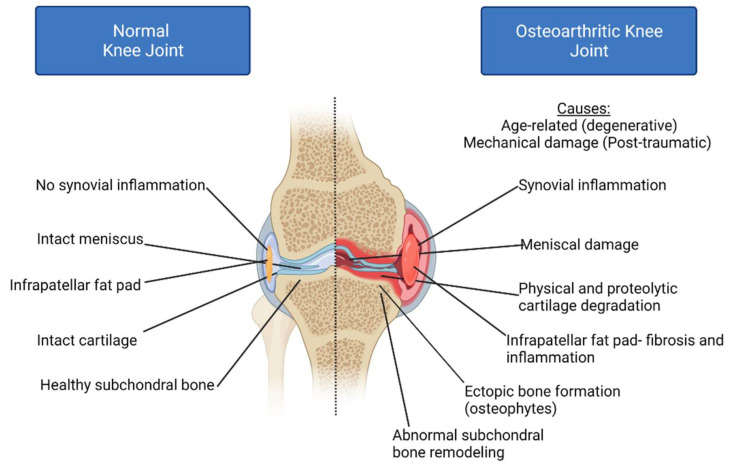
Osteoarthritis pathophysiology: Osteoarthritis (OA) is a multifactorial disease affecting cartilage, synovium, the infrapatellar fat pad, and the underlying subchondral bone. OA is characterized by chronic inflammation, cartilage damage due to mechanical and proteolytic degradation and abnormal subchondral bone formation, leading to the formation of bony outgrowths into the joint capsule referred to as osteophytes.

The development and progression of osteoarthritis (OA) are also negatively affected by epigenetic dysregulation. OA is a debilitating joint disease and instigator of chronic disability that affects approximately half of the global population over the age of 65, incurring a substantial socioeconomic burden (USD 128 billion in 2003 alone and rising) [13,14,15,16]. The prevalence of this disease is expected to continue to grow as global life expectancies rise [17]. OA pathophysiology is most associated with synovitis (chronic synovial inflammation), ectopic new bone formation (osteophytes), abnormal subchondral bone remodeling, and the degradation of articular cartilage due to physical and proteolytic degradation [18,19,20] (Figure 1). However, OA is a multifactorial disease that also affects the meniscus, ligament structures, and infrapatellar fat pad, meaning that it is a whole joint disease [21,22]. Known risk factors for the disease include microenvironment and mechanical stressors, physical activity levels, health status, obesity, genetic predisposition, and age [23,24,25]. One of the biggest difficulties in the understanding and treatment of the disease is that OA is symptomatic only in approximately 30% of patients in later disease stages, making the detection of early biomarkers difficult [26,27]. At present, no disease-modifying drugs are available to decelerate or reverse OA. Current OA treatments are limited to pharmacological pain management and arthroplasty, an irreversible surgical procedure that typically requires revisional surgery in 10–15 years [28,29].

One of the biggest problems in the treatment of OA is that the affected tissue, articular cartilage, is unable to regenerate to repair mechanical or enzymatic damage. Articular chondrocytes, the resident cell type present in cartilage, synthesize an extracellular matrix (ECM) capable of withstanding biomechanical pressures and responsible for smooth joint articulation. Under normal conditions, chondrocytes are quiescent and catabolic and anabolic factors are balanced to maintain joint homeostasis. When damage occurs, chondrocytes cannot regenerate the damaged tissue and actively participate in cartilage destruction by enhancing the production of catabolic proteases [21,30]. The molecular basis for this switch in chondrocyte function remains poorly understood, but aberrant gene expression and epigenetic modifications are implicated [31].

OA is known to have genetic components associated with disease onset and progression with dozens of loci associated with the disease [32]. These loci alter gene expression and contribute to OA by either altering gene expression patterns or through post-translational modifications [33]. Chondrocyte gene expression is regulated by a variety of epigenetic mechanisms including the methylation of DNA and histones, histone acetylation, ncRNAs and polycomb group proteins (PRCs) [34]. In this review, we will discuss these epigenetic mechanisms and how they contribute to the onset and progression of OA.

## 2. Methods

A PUBMED literature review was conducted searching for original papers on epigenetic regulation in OA. The key words used included “DNA methylation in osteoarthritis”, “epigenetics and osteoarthritis”, “epigenetics and synovium in osteoarthritis”, “histone modification and osteoarthritis”, “polycomb and osteoarthritis”, “miRNA and osteoarthritis”, “siRNA and osteoarthritis”, “non-coding RNA and osteoarthritis”, “epigenetic regulation of subchondral bone”, “osteoarthritis therapeutics”, and “epigenetic therapies and osteoarthritis”.

## 3. DNA Methylation

DNA methylation involves the addition of a methyl group to a specific location along the DNA strand. This reaction is catalyzed by DNA methyltransferases (DNMTs) and there are currently four known mammalian enzymes: DNMT1, DNMT3a, DNMT3b and DNMT3L [35,36]. The biological importance of this addition is that it alters the three-dimensional conformation of the DNA and, depending on the location, can either enhance or inhibit the ability of transcription factors and/or associated proteins to bind DNA [37,38]. In OA, changes in DNA methylation patterns are some of the most widely studied epigenetic phenomenon.

Early studies of DNA methylation patterns associated with OA were targeted primarily on candidate genes already known to be associated with OA pathophysiology. Candidates often included catabolic cytokines and chemokines such as the matrix metalloproteinases (MMP-3, MMP-9, and MMP-13) and aggrecanases A disintegrin and metalloproteinase with thrombospondin motifs 4 and 5 (ADAMTS-4 and ADAMTS-5) that are upregulated in OA and contribute to proteolytic cartilage degradation [39,40]. Proinflammatory molecules, such as interleukins (IL-1β, IL-6 and IL-8), inducible nitric oxide synthase (iNOS) and tumor necrosis factor-alpha (TNF-α), responsible for the initiation and maintenance of chronic joint inflammation were also targeted [41,42]. The results from these studies determined that the promoters of these catabolic factors are demethylated in OA, permitting increased catabolic gene expression [34,43,44,45]. Anabolic factors known to be downregulated in OA pathogenesis, such as cartilage ECM genes type-II collagen (Col2α1) and aggrecan (ACAN) were targeted as well and found to be hypermethylated, inhibiting their expression [46,47]. Studies into DNA methylation changes during OA have also focused on signaling pathways known to be altered in articular cartilage and subchondral bone during OA. For instance, the Wnt pathway is known to be upregulated during OA progression and epigenetic changes have been detected that altered sclerostin (SOST) and Wnt-ligand secretion mediator (WLS) function to increase inflammation and endochondral ossification, respectively [41,48].

More recently, OA DNA methylation studies have switched to non-targeted genome-wide methylome studies to identify novel genes or regions affected by the disease. The standardization techniques used in these non-targeted genome-wide assays make comparisons between datasets easier to manage and promote more collaborative studies, which increase sample sizes and allow for examinations of OA-specific DNA methylation changes at various stages of disease progression and analyses of global datasets. Analyses of these global datasets are beginning to detect regional and/or ethnic methylations that contribute to disease susceptibility. For instance, meta-analyses of single-nucleotide polymorphisms (SNPs) of European, Japanese and East Asian populations have identified region-specific SNPs in type-11α1 collagen (COL11α1), vascular endothelial growth factor (VEGF) and growth differentiation factor 5 (GDF5) that enhance susceptibility to OA [40,49,50,51,52,53,54]. Furthermore, these large-scale analyses can detect sex-specific and location-specific differences in susceptibility loci to better predict predilection to hip versus knee OA within a population [40,55,56,57]. Future studies identifying OA-specific SNPs and loci differences within populations will continue to improve our knowledge of OA-affected genetic differences and will increase risk prediction.

## 4. Histone Methylation

**Figure 2:**
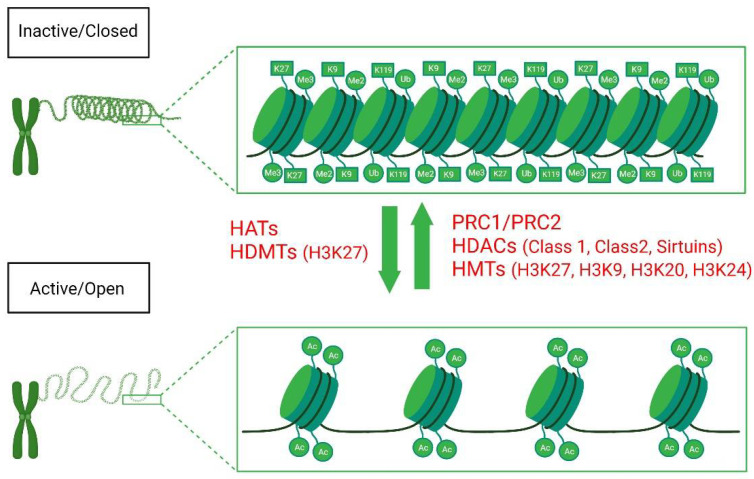
Histone modifications in OA: Histone methylation/demethylation and histone acetylation/deacetylation affect genetics architecture and the accessibility of transcriptional activity. The process of histone methylation is governed by histone methyltransferases (HMTs) and histone demethyltransferases (HDMTs). Histone acetylation involves the addition or the removal of an acetyl group by histone acetyltransferases (HATs) or histone deacetylases (HDACs), respectively.

Genetic architecture plays and enormous role in transcriptional control. The structure and accessibility of DNA for transcriptional activity are partly governed by the location and function of histones, conserved regions of DNA enriched with positively charged amino acids such as lysine (K) and arginine (R) [41,58]. These positively charged regions interact tightly with the negatively charged DNA backbone and accessory DNA binding proteins to form tightly wound regions called nucleosomes [58,59]. This winding makes the DNA inaccessible, which protects it from damage and regulates the transcriptional control of the affected regions [58,60]. An additional level of control governing histone-DNA interactions are post-transcriptional modifications to the side chains of the amino acids comprising the histone. The various modifications known to occur include sumoylation (the covalent attachment of small ubiquitin-like modifiers (SUMO) to lysine residues), phosphorylation, ubiquitination (the covalent attachment of ubiquitin to lysine residues), and poly (ADP)-ribosylation [61,62,63,64,65]. The two most widely known and widely studied post-transcriptional modifications in the pathogenesis of OA, however, are histone methylation and histone acetylation (Figure 2). The process of histone methylation and its relevance to the pathophysiology of OA will be discussed here and histone acetylation in the following subsection.

Histones are methylated by histone methyltransferases (HMTs) that catalyze the addition of a methyl group to specific arginine or lysine residues on histone tail side chains [41,65]. This reaction is reversible and the added methyl groups can be removed through the actions of histone demethyltransferases (HDMTs). Unlike DNA methylation, the effects of histone methylation depend highly on the site of methylation as well as the degree of methylation (mono-, di- or trimethylation) [65,66]. Some of the most well-studied sites for histone methylation occur on lysine residues associated with histone 3 (H3) [65,67,68]. For example, the inhibition of HMT activity at H3 lysine 4 (H3K4) reduced inflammation by preventing iNOS and cyclooxygenase-2 (COX-2) expression in chondrocytes, and methylation at lysines 9, 20 and 24 (H3K9, H3K20 and H3K24) are known to be altered in OA [38,69,70,71,72]. Histone methylation changes are also known to affect the expression of SOX-9 (SRY-box transcription factor-9), an important transcription factor regulating COL2α1 expression. SOX-9 expression is downregulated in OA and studies of epigenetic modifiers have linked SOX-9 inhibition to the increased methylation of H3K27 [46,73,74,75]. Furthermore, the methylation of the NFATC1 (Nuclear Factor of Activated T-cells-1) promoter has been shown to be increased in OA and contributes to chondrocyte dysfunction [76,77].

Perhaps one of the best examples of the role of HMTs in OA pathology is that of DOT1-like histone lysine methyltransferase (DOT1L). DOT1L was one of the first methyltransferases identified as associated with OA and subsequent studies have shed additional light on the protective role of this enzyme in cartilage and chondrocyte biology [78]. DOT1L is the only known mammalian HMT to catalyze the methylation of H3 lysine79 (H3K79) [79]. The protective mechanism of DOT1L is two-fold: it inhibits sirtuin 1 (SIRT1), a highly conserved deacetylase that contributes to OA progression, and also methylates promoters of lymphoid enhancer-binding factor 1 (LEF1) and T-cell factor 1 (TCF1) to modulate wingless-type (Wnt) signaling in chondrocytes [80,81]. In patients suffering from OA, H3K79 methylation was reduced, resulting in Wnt pathway activation and increased cartilage degradation in humans as well as in murine OA models [82,83].

KDM6B (JMJD3) is emerging as a demethylase of interest in the onset and progression of OA. KDM6B catalyzes the removal of methyl groups from histone 3 lysine 27 (H3K27) and functions to modulate cartilage anabolism and homeostasis [69,84]. In chondrocytes, the knockdown of KDM6B in mice results in abnormal bone and cartilage development and accelerates the progression of OA [84,85]. Furthermore, KDM6B is associated with chondrocyte hypertrophy by increasing Runt-related transcription factor 2 (RUNX2) and Indian hedgehog (IHH) signaling [85,86]. The overactivation of RUNX2 also negatively impacts OA pathogenesis by enhancing subchondral bone ossification and remodeling [84,85].

## 5. Histone Acetylation

Similar to histone methylation, dysregulated histone acetylation/deacetylation is known to play a role in enhanced proinflammatory cytokine and chemokine activity in both rheumatoid and osteoarthritis [87,88]. The addition or removal of an acetyl group from histones affects gene transcription and accessibility (Figure 2). Histone acetyltransferases (HATs) catalyze the addition of an acetyl group to a histone or histone sidechain, which loosens DNA-histone binding and enhances gene transcription [89]. Conversely, the removal of acetyl groups is catalyzed by histone deacetylases (HDACs) that are recruited by transcription factors and protein complexes to enhance the binding between histones and DNA to silence transcription [87,90]. Additionally, both HATs and HDACs by themselves are known to interact with and destabilize non-histone-associated proteins and co-factors, leading to cellular dysfunction [91,92]. Unfortunately, this interaction with additional accessory proteins and/or transcription factors outside of DNA–histone interactions places limitations on the use of HAT and HDAC knockout animal models [18,93].

Of these reactions, much more is known about HDACs and their role in the development and progression of OA. HDACs comprise four separate classes divided by enzymatic structure, substrate usage, function, and location. Class I (1, 2, 3 and 8) and Class II (4, 5, 6, 7, 9 and 10) HDACs use zinc as a common substrate, while Class III HDACs, also referred to as sirtuins (Sirt 1-7), utilize nicotinamide adenine dinucleotides (NAD^+^) [87,94,95]. Class I enzymes are vital for cell survival and regulate gene transcription and DNA replication during development [96,97]. Knockout models of these are embryonically lethal. Class II enzymes have weaker enzymatic activity but are more tissue specific in their function [98,99]. Class III enzymes (the Sirts) possess the highest enzymatic activity of the HDACs and studies have shown that their activity affects a multitude of different pathways including (but not limited to) aging, inflammation, bone formation, maintenance, and metabolism [100,101].

Several studies have examined the role of HDACs in osteoarthritis. During OA development and progression, the activity of matrix catabolic factors is elevated, while anabolic factors are suppressed. Recently, studies on diseased cartilage have linked HDAC activity to reductions in anabolic factors. For instance, OA chondrocytes were found to have elevated levels of HDACs 1 and 2 which can inhibit the expression of key anabolic factors Col2α1 and ACAN [89,102,103]. In vitro studies have shown that Runx2, known to stimulate chondrocyte hypertrophy and MMP-13 production, is inhibited by HDAC4 but this interaction requires better study in vivo prior to therapeutic assessment [104,105,106]. HDAC7 is elevated in diseased OA cartilage and studies of the mechanism in murine models have linked it to elevated MMP-13 expression [107].

The expression of some HDACs, however, is known to have chondroprotective properties. SIRT6, for instance, plays a protective role in human and murine chondrocytes. In studies of human OA, SIRT6 expression is decreased in articular cartilage, and the overexpression of Sirt6 in mice reduces cartilage damage and the expression of proinflammatory cytokines and chemokines [108]. SIRT1, linked to increased Col2α1 expression, is also related to protective functions and SIRT1 expression is known to decline during OA in human chondrocytes and in subchondral osteoblasts [109,110]. Finally, the continuing development of knockout mouse models of HDACs 3-5 and HDAC7 allows for the continuation of more focused studies of endochondral bone formation and the changes that occur with age-related or induced post-traumatic osteoarthritis [111,112].

HDAC activity in OA is not limited to chondrocytes and cartilage. Synovial tissues are known to contribute to chronic joint inflammation through the secretion of proinflammatory cytokines and chemokines such as IL-6, IL-1β and TNF-α [113]. The best known function of IL-6 in OA is proinflammatory, but IL-6 also stimulates MMP-13 expression and activates osteoclast activity in subchondral bone, enhancing skeletal remodeling [114]. Epigenetic studies into IL-6 expression in the synovium of OA demonstrated that the promoter region of IL-6 is hypo-methylated, and the promoter histone region is hyper-acetylated, leading to increased IL-6 production [115]. While there are fewer studies on the epigenetic regulation of synovial tissues in OA, these findings demonstrate that the modulation of synovial epigenetics may be of interest for future studies towards pharmacological interventions.

## 6. Polycomb Repressive Complexes (PRCs)

**Figure 3:**
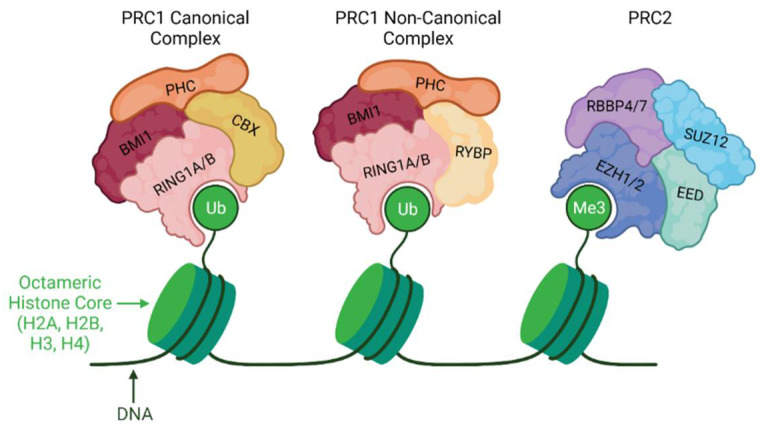
Polycomb Group Complexes (PRCs) in OA: Polycomb repressive complex 1 (PRC1) and Polycomb repressive complex 2 (PRC2) are protein complexes that contribute to chromatin compaction to regulate development, cell proliferation, and differentiation. In OA, dysregulation of PRCs contributes to increased inflammation and aberrant Wnt pathway signaling activation, resulting in premature chondrocytes differentiation and accelerated chondrocyte hypertrophy differentiation.

Polycomb repressive complexes (PRCs) are key enzymes for regulating and modifying chromatin structure and histones. Polycomb repressive complex 1 (PRC1) and Polycomb repressive complex 2 (PRC2) are the two main repressive PcG protein complexes that contribute to chromatin compaction and play a crucial role during development, cell proliferation, and differentiation (Figure 3) [116,117]. PRCs are important in the silencing of genes globally, particularly during mitotic cycles that can act as regulatory mechanisms. Additionally, PRC proteins are biologically essential throughout development from embryogenesis to adulthood, particularly in the regulation of imprinted genes [118,119,120].

PRC1, composed of the subunits BMI1, PHC, CBX, and RING1A/B, is involved in transcriptional repression through the ubiquitination of histone 2 lysine 119 (H2K119) [121,122,123]. PRC2 is a chromatin-modifying enzyme that catalyzes the trimethylation of histone H3 at lysine 27 (H3K27), which regulates gene expression [124]. PRC2 contains four core subunits, SUZ12, EED, EZH1/EZH2, and RBBP4/7, which form two functional lobes [125,126]. The catalytic lobe that methylates H3K27 is comprised of the methyltransferase subunits enhancer of Zeste 1 polycomb repressive complex subunit-1 (EZH1) or enhancer of Zeste 2 polycomb repressive complex subunit-2 (EZH2), a binding protein called embryonic ectoderm development (EED), and the scaffold protein Zeste 12 homolog (SUZ12) [127,128]. The targeting lobe consists of SUZ12 and RBBP4/7 [125,129].

Studies into the epigenetic regulation of PRCs in the pathogenesis of human OA have recently paid particular attention to the importance of EZH2 in the development and progression of the disease. EZH2 has been found to be upregulated in both cartilage and chondrocytes of human OA patients [130,131] and in the cartilage of mice following the induction of post-traumatic OA via the destabilization of the medial meniscus (DMM) surgery [130,132,133]. This upregulation of EZH2 results in the enhanced expression of proinflammatory cytokines/chemokines (IL-1β, nitric oxide (NO) and IL-6), increased ECM catabolism via increased activity of MMPs and mediates Wnt pathway inhibitor-secreted frizzle-related protein (SFRP-1) [131,134]. While these findings seem to suggest that the EZH2 inhibition in cartilage would be undoubtedly beneficial, other studies have pointed out that the timing of ablation is important to the maintenance of chondrocyte homeostasis. A tissue-specific mouse EZH2 knockout mouse model showed enhanced cartilage destruction following the induction of post-traumatic OA via medial meniscectomy (MMx) surgery, suggesting that EZH2 plays a selective chondroprotective role [135]. A conditional knockout (cKO) of EZH2 in mouse mesenchymal stem cells resulted in a smaller body size and shorter limbs [136]. A histological analysis of the proximal tibias of one day and three-week-old EZH2 cKO pups revealed an abnormality in growth plate development, which was represented in the reduced distance between the surface of articular cartilage and the hypertrophic zone [136]. A reduction in distance between epiphysis and the hypertrophic zone is associated with a shorter proliferative area in the one-day-old EZH2 cKO pups and a reduced hypertrophic zone in the three-week-old EZH2 cKO mice. The importance of EZH2 function in chondrocytes and growth plate development is indicated by the reduced chondrocytes proliferation and accelerated hypertrophy in EZH2 cKO mice [136]. More studies are needed to assess the true role of EZH2 in OA pathophysiology.

PRC2 maintains gene transcription through alterations in cellular genetic makeup that govern normal cellular development. PRC2 is modulated by accessory subunits as well as various modifications to either stimulate or inhibit its activity on H3K27. Histone modifications and dense chromatin stimulate PRC2, while active chromatin inhibits it to prevent the spreading of H3K27 methylation [137,138]. PRC2 is necessary for normal skeletal and cartilage growth. When certain subunits of PRC2 are deficient, cartilaginous abnormalities can occur. For example, when EED is deficient, there have been reports that there is aberrant signaling activation in the Wnt pathway which causes premature differentiation, ultimately leading to kyphosis and accelerated chondrocyte hypertrophic differentiation [139]. In addition, the overactivation of TGF-*β* led to a reduction in chondrocyte proliferation with growth defects [140]. The functional activity of Z hypoxia-inducible transcription factor 1*α* (Hif1*α*) is required to maintain chondrocyte viability within the central region of the growth plate, and EED deficiency decreases Hif1*α*, ultimately leading to hypoxic cell death [139].

## 7. Non-Coding RNAs (ncRNAs)

Only approximately 1.2% of the human genome consists of coding regions, indicating the profound role and potential utility of non-coding regions [141]. Non-coding RNA (ncRNA) is a term applied to RNAs that do not code for proteins and that are sometimes thought of as “junk RNA” [142]. However, some ncRNAs have been known to have biological functions for decades, with early studies identifying roles in the regulation of chromosomes compaction (Xist), and in cell-type specific nuclear organization [143,144,145]. Further studies have supported the role of ncRNAs in epigenetic regulation and the remodeling of chromatin, although these roles are still being debated [146,147]. The term ncRNA includes a wide range of molecules such as small interfering RNAs (siRNAs) microRNAs (miRNAs), small nucleolar RNAs (snoRNAs), small nuclear RNAs (snRNAs), long non-coding RNAs (lncRNAs), and, more recently, circular RNAs (circRNAs), all of which have been implicated in the pathogenesis of OA [148,149,150].

**Figure 4:**
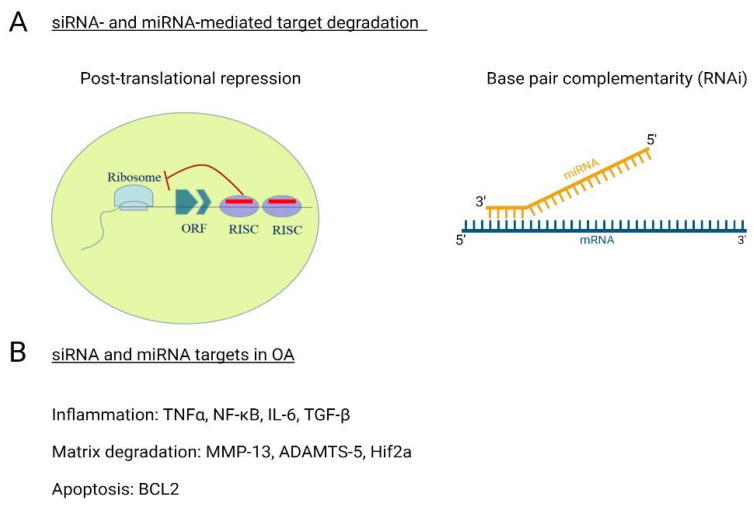
siRNA- and miRNA-targeted repression in OA. Small interfering RNAs (siRNAs) and microRNAs (miRNAs) and two types of non-coding RNAs (ncRNAS) that alter gene expression patterns via (**A**) post-translational repression or base pair complementarity (RNAi). siRNAs and miRNAs are known to target various aspects of OA pathology including (**B**) inflammation of chondrocytes and synovium, matrix degradation (enhanced enzymatic production), matrix anabolism (inhibition of anabolic expression) and chondrocyte apoptosis. Red line indicates siRNA or miRNA inclusion in the RNA-induced silencing complex (RISC).

siRNAs are double stranded, non-coding RNAs approximately 21–23 nucleotides in length (Figure 4) that typically have one specific target [151]. siRNAs are known to protect the genome from exogenous or invasive nucleic acids, such as viruses and transposons [152]. siRNAs can induce target degradation after translation through the formation of an RNA-induced silencing complex (RISC) or directly target mRNAs for degradation through base pair complementarity (RNAi) [152,153]. The role of siRNAs in the onset and development of OA is ever expanding. Given that OA is a polygenic disease, researchers typically focus the siRNA discovery of chondrocytes, cartilage, and synovium on one aspect, such as chronic inflammation or matrix degradation. For instance, the siRNA-mediated knockdown of ADAMTS-5 and MMP-13 through intra-articular injection ameliorated cartilage damage and inflammation following DMM in mice [154,155]. Similar findings were reported when siRNA targeting MMP-13 were delivered using intra-articular nanoparticle delivery methods [156]. RNA-silencing was also found to be effective at inhibiting inflammation through the targeting of TNFα and Transforming growth factorβ-activated kinase-1 (TAK1) in a collagen-induced murine models [157]. These findings demonstrate the efficacy of siRNA-mediated targeting strategies in the treatment of OA, but further work is needed to validate these findings in human joints. Meanwhile, other key targets for siRNA-mediated inhibition include the NF-κB and transforming growth factor-beta (TGF-β) pathways (inflammation), SOST (subchondral bone remodeling), hypoxia-induced factor 2a (Hif2a) (cartilage degeneration and synovitis), MMP13 (matrix catabolism) and mitochondrial dysfunction, which results in cell apoptosis [158,159,160].

Mature miRNAs are non-coding, single stranded and range from 21–24 nucleotides in length (Figure 4). miRNAs regulate gene expression through the targeting and cleavage of mRNA or via translational repression through binding interactions with the 3′untranslated region (3′ UTR) of target mRNAs [161,162]. New miRNAs are being discovered regularly and the function of these new biomolecules is under study. Currently, miRNAs are known to be involved in the regulation of cell differentiation, cell cycle progression, apoptosis, lipid metabolism, gene and protein expression and the modulation of intercellular communication in numerous cell types [163,164,165,166,167]. Given the diversity of their biological roles, it is unsurprising that aberrant miRNA expression has been linked to OA and there are many reviews that focus solely on these biomolecules. Here, we will provide a brief overview of the role of miRNAs in OA pathogenesis to provide insight into their epigenetic function (Table 1). One early study into the role of miRNAs in OA pathogenesis examined the expression patterns of miRNAs in knee cartilage and bone samples of arthroplasty patients compared cartilage from to post-mortem healthy cartilage and bone [168]. This study identified 157 miRNAs that were statistically different in the OA individuals and two specific miRNAs, miR-9 and miR-98, were subsequently identified to upregulate the expression MMP-13, IL-6 and TNFα [168,169]. miRNA-140 is another miRNA with functional roles in healthy and diseased chondrocytes. miRNA-140 is highly expressed during endochondral ossification of long bones and significantly decreased under inflammatory conditions in vitro and in OA tissues in vivo [170,171,172]. The functional role of miRNA-140 was discovered to be the inhibition of ADAMTS-5 aggrecanase expression as well as the post-translational inhibition of MMP-13 and insulin-like growth factor binding protein 5 (IGFBP-5) [171,172,173,174]. miRNAs are also capable of regulating chondrocyte anabolic expression. miRNA-148a serves dual chondroprotective roles: it inhibits chondrocyte hypertrophy and enhances Col2α1 chondrocyte deposition [175]. Recent findings demonstrate that miR-4784 enhances Col2α1 expression while simultaneously inhibiting MMP-3 to reduce ECM degradation [176,177]. Similarly, the miRNAs miR-98 and miR-181a play a role in chondrocyte homeostasis through the inhibition of BCL2 apoptosis regulator (BCL2) translation to slow the accelerated chondrocyte apoptosis seen in OA [178,179]. Conversely, miR-101 and miR-145 upregulate ECM catabolism and, when inhibited, lead to increases in SOX-9, Col2α1 and proteoglycan deposition [180].

miRNAs have also been discovered to contribute to OA synovial pathology. Examinations of synovium from a murine DMM model identified 394 differentially expressed miRNAs during post-traumatic OA development [181]. Additionally, studies of miRNA expression in human OA detected differential expression in disease chondrocytes and cartilage. These were found to have various roles in disease development such as increased inflammatory response (miRs-381a-3p, 34a, 146a, 181a), increased NF-κB signaling (miR-381a-3p), and enhanced angiogenesis (miR-125) [182,183,184,185,186]. Synovial miRNAs can also serve anti-inflammatory functions and protect against ECM degradation [187,188,189]. While many studies have focused on the miRNA contributions in chondrocyte and cartilage pathology, fewer have focused on synovial miRNA expression. Given the importance of the synovium in the onset and progression of OA, more studies are needed to fully understand the contribution of synovial miRNAs in OA and their mechanisms of action.

**Figure 5:**
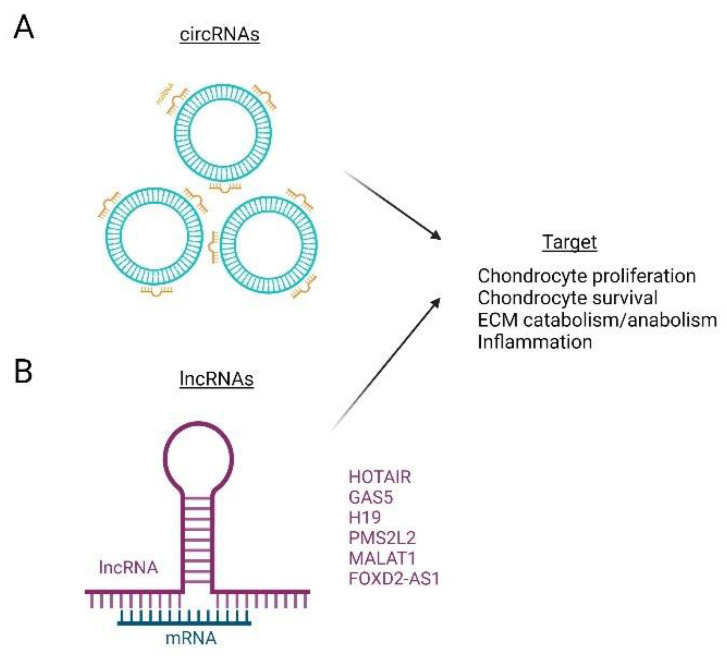
circRNA- and lncRNA-targeted modulation in OA. circRNAs (**A**) and long non-coding RNAs (lncRNAs) (**B**) contribute to disease pathogenesis in OA through the dysregulation of chondrocyte proliferation and survival, altered anabolism and catabolism of the extracellular matrix (ECM) and through changes in inflammation.

Long non-coding RNAs (lncRNAs) are non-coding RNAs classified by their size, being greater than 200 nucleotides in length (Figure 5). lncRNAs play a role in cellular structure integrity, transcription, splicing, translation, protein localization, cell cycle, apoptosis, stem cell pluripotency, embryonic development, immune responses and more [149,190]. Less is known about the role of lncRNAs in skeletal biology, but aberrant lncRNA expression has been implicated in the development of cancers, cardiovascular and neurodegenerative diseases and inflammatory diseases, such as OA [191,192,193,194,195]. lncRNAs, like other ncRNAs, have been linked to various aspects of OA such as chondrocyte apoptosis, ECM degradation and the maintenance of the chronic inflammatory response [191,196,197]. For example, the upregulation of lncRNAs such as HOTAIR, GAS5, H19, PMS2L2, and others, may increase mRNA expression for bone morphogenic protein-2 (BMP-2), ADAMTS5, MMP-9, and MMP-13 [198,199,200]. Metastasis-associated lung adenocarcinoma transcript 1 (MALAT1) is also associated with OA. MALAT1 expression is upregulated in OA cartilage and results in increased chondrocyte proliferation and the inhibition of apoptosis [201,202]. Another lncRNA, FOXD2-adjeacent opposite strand RNA 1 (FOXD2-AS1), is also upregulated in OA and enhances ECM degradation in OA via increased Toll-like receptor 4 (TRL4) expression [197,203]. A study examining differences in OA knee arthroplasty lncRNA expression identified 3007 lncRNAs were upregulated and 1707 were downregulated in comparison to normal samples [204]. These numbers indicate the breadth of lncRNA involvement in OA, for which further investigation is required.

circRNAs are novel ncRNAs that have evaded recognition until recently for a variety of reasons (Figure 5). circRNAs are separated from other RNAs by their circular shape, not by their size, and they have no definitive 3′ or 5′ ends for techniques recognizing the free RNA ends to acknowledge [205]. circRNAs are very stable molecules with half-lives of approximately 48 h [206]. circRNAs can be exonic or intronic, can be translated, and can influence transcriptional regulation through the regulation of miRNA [207]. These ncRNAs are involved in the pathogenesis of diseases such as diabetes, cancer, cardiovascular disease and OA [208]. In OA, circRNAs are known effect the proliferation and survival of chondrocytes as well as mediate cartilage metabolism and inflammation within the joint [208,209,210,211]. circRNAs often function as miRNA sponges, downregulating the expression of aggrecan and COL2 and enhancing the expression of ADAMTS-4, MMP-3, and MMP-13, thus leading to cartilage degradation [150,208]. Via miRNAs’ sponge activity, circRNAs have also been shown to lead to the degradation of the cartilage matrix [208]. However, circRNAs such as circSERPINE2 have been shown to have anabolic properties and may have the potential to be protective against OA [212]. Similarly, circCDK14 has been shown to protect ECM composition during OA by inhibiting Smad2 expression and inhibiting TGF-β signaling in chondrocytes [213].

Recent work into the mechanisms of ncRNA function have also identified that various ncRNAs can interact through a competitive endogenous RNA (ceRNA) axis that can alter the speed and severity of OA progression [214]. Through this interaction, ncRNAs can regulate the timing and expression of other ncRNA to alter cell homeostasis and disease pathology [215]. This novel pathway of cellular regulation is still poorly understood and warrants future study.

**Table 1:** ncRNAs.

ncRNA	OA Function	References
miR-9	Enhance ECM degradation	[171,172]
miR-98	Enhance ECM degradation	[171,172]
miR-140	Inhibit ECM degradation	[173,174,175,176,177]
miR-148a	Enhanced ECM anabolism Inhibit hypertrophy	[178]
miR-4784	Enhances ECM anabolism Inhibit ECM degradation	[179,180]
miR-98	Inhibit ECM degradation Inhibit inflammation	[171]
miR-181a	Inhibit ECM degradation Inhibit inflammation	[171]
miR-101	Enhance ECM degradation	[183]
miR-145	Enhance ECM degradation	[183]
miR-381a-3p	Proinflammatory	[185,186,187,188,189]
miR-34a	Proinflammatory	[185,186,187,188,189]
miR-146a	Proinflammatory	[185,186,187,188,189]
miR-181a	Proinflammatory	[185,186,187,188,189]
circCDK14	Inhibit ECM degradation	[205]
miR-125	Increased angiogenesis	[185,186,187,188,189]
HOTAIR	Enhance ECM degradation	[203]
GAS5	Proinflammatory	[202]
H19	Proinflammatory	[201]
MALAT1	Inhibit apoptosis Increase proliferation	[204,205]
FOXD2-AS1	Enhance ECM degradation	[206,207]

## 8. Epigenetics of Obesity

The prevalence of obesity in the general population is increasing and obesity itself is now identified as a global health problem [216]. The link between weight, physical activity levels, and OA is well established. Mechanical stressors such as joint loading are increased in obese individuals and are thought to contribute to disease onset and progression [217,218]. To help combat this process, weight loss is one lifestyle alteration commonly suggested in OA patients to reduce joint stress and stiffness [219,220,221].

However, it is not just weight itself that forms such a strong link between weight and OA. Adipose tissue is an active endocrine organ that secretes cytokines as well as adipokines, adipose-secreted proteins [222]. These secreted factors have systemic and local functions and influence insulin resistance, vascular function, renal function, β cell function, and cancer [223,224,225,226,227]. In OA, adipokines such as leptin, adiponectin, resistin, visfatin, and chemerin have been shown to be differentially expressed in the cartilage, synovium, and/or infrapatellar fat pads of OA patients and mechanistic studies have linked these molecules to cartilage and synovial inflammation, increased chondrocyte catabolism, and increased joint pain [228,229,230,231,232,233,234].

Recent studies have begun to examine the epigenetic regulation of obesity and the link with OA. For instance, the expression of two microRNAs (miR-935 and miR-4772) are statistically increased in obese patients [235]. Examinations of miRNA profiles in patients who have lost weight either by lifestyle changes or bariatric surgery have identified alterations in miRNA profiles post weight loss [236]. These findings have led some individuals to target anti-miRNA therapies for OA. Preclinical studies are now underway with several therapies showing potential [237]. One example of this is miR-146a that suppresses MMP-13 and ADAMTS-5 following IL-1 proinflammatory stimulation [238].

Other studies have examined alternative epigenetic regulators of obesity. Altered DNA and histone methylation patterns have been identified in obese individuals suffering from vascular disease, diabetes, and energy metabolism [239,240,241]. These findings, and the increasing body of work detailing age-related epigenetic changes, lend increasing support that epigenetic dysregulation contributes to OA pathology and highlight the need for further studies [242,243,244].

## 9. Epigenetic Regulators as Therapeutic Options

The development of therapeutic treatments to slow or prevent OA is challenging due to its multifactorial genetic involvement, the involvement of numerous tissue types, incomplete pathogenesis, and disease heterogeneity [158,245,246]. Pharmacological treatment options under development and evaluation seek to mitigate disease-associated pain and progression by targeting key proinflammatory mediators. Recent developments in the field of OA epigenetic regulation have also yielded new potential targets for OA therapeutics.

Traditionally, therapeutics have targeted the molecular pathways known to be affected in OA pathogenesis. For instance, cartilage degradation due to proteolytic enzymes have been countered with metalloproteinase and aggrecanase inhibitors and bisphosphonates have been prescribed to rebalance bone homeostasis (2). However, these therapies have had limited success due to differences in patient genetic backgrounds and the mixed effects between different joint tissues. These findings highlight the potential use of epigenetic targets for OA treatments, particularly epigenetic regulators found in all joint cell and tissue types.

One therapeutic target that shows promise is ten-eleven-translocation (TET) enzymes TET1, -2 and -3 methylcytosine dioxygenases function in DNA hydroxymethylation and have been shown to have cell-specific gene expression regulatory functions [247,248,249]. Additionally, TETs have been shown to regulate multiple OA-related anabolic (Col2α1, Acan) and catabolic (Mmp-3, Mmp-13 and Adamts-5) genes [250,251,252]. TETs are also involved in dysregulated chondrocyte homeostasis through the regulation of the Wnt and mTOR pathways [252]. A pharmacological inhibitor, 2-hydroxyglutarate, has been shown to inhibit OA development in a murine destabilization of the medial meniscus model highlighting the therapeutic potential of the molecule, but this has yet to be investigated in clinical trials [252,253]. Other molecules have also been developed that target DNA methyltransferases (DMTs) activity such as TETs. Two inhibitors, azacytidine and decitabine, have already been granted FDA approval for use in blood, colorectal, ovarian and breast cancers [231,254,255,256,257]. Based on in vitro studies, decitabine also shows potential for use in OA but no clinical trials are presently examining this role [258].

The pharmacological inhibition of polycomb recessive complexes is also currently under investigation as potential therapeutics. A small-molecule inhibitor of PRC2 complex member EZH2, EPZ005687, is under evaluation in murine OA models. The results have been mixed. Using pharmacological inhibition with this molecule, OA was inhibited following induced medial meniscectomy surgery [134]. However, the genetic ablation of EZH2 in a cartilage-specific murine model showed exacerbated OA development [135]. These findings highlight the potential of EZH2 pharmacological intervention, but caution that patient genetic background, cell-type-specific events and timing need to be further studied. Finally, other EZH2 inhibitors (such as tazemetostat, E7438, GSK126 and UNC1999) have been utilized in various cancer treatments, and it would be beneficial to test these drugs for efficacy in OA [259,260,261,262].

Histone deacetlyases (HDACs) have been a potential therapeutic option for many years now. The inhibition of these enzymes through HDACi shows potential for the inhibition of matrix catabolism and inflammation. However, an early HDAC inhibitor, Trichostatin A, failed to inhibit MMP-13 expression in human chondrocytes [107,263,264]. Interestingly, the siRNA-mediated inhibition of HDAC7 did inhibit MMP-13, demonstrating that the inhibition of specific HDACs, versus a broad-range inhibitor such as Trichostatin A, may be a more beneficial approach [107]. This hypothesis was further validated when HDAC4 inhibition with SW1353 inhibited inflammation following proinflammatory stimulation [104,265]. While continuing studies of the long-term usage of these drugs suggests HDACis have detrimental effects on the skeleton in children and adults, HDAC inhibitors have been found to be largely beneficial to the health of chondrocytes [89,266,267]. One Class III HDAC, Sirt1, has been shown to stabilize chondrocyte homeostasis and demonstrates reduced expression in OA patients [268,269,270]. Sirt1 activator, resvertol, has recently been shown to protect against cartilage damage in surgical OA murine and rat models, but human trials are still needed to test the effects in human OA [271,272,273]. To further our knowledge about HDACi in OA and towards the development of new therapeutic approaches, other specific HDAC inhibitors, including ACY-1215, RGFP966 and vorinostat, are being used in ongoing clinical and murine trials to evaluate the efficacy of these molecules alone and in combination [266,274,275].

Cell therapy is another therapeutic option gaining favor in the treatment of OA. Studies of other disease pathologies have identified that mesenchymal stem cells (MSCs) can interact with the immune system and modulate aspects of the anti-inflammatory response [276,277,278], making them of interest in the treatment of OA. Preclinical studies of intra-articular injections showed promise in animal preclinical trials and researchers are developing various biological packaging options to minimize the risks of injection site leakage and the possibility of rejection effects [279,280]. At present, several clinical trials are underway in the United States to test the efficacy of intra-articular injections of MSCs, bone marrow aspirates and platelet-rich plasma in OA treatment.

Currently no siRNA therapeutic agents are on the market or in clinical trials in the treatment of OA; however, they are on the market for other conditions such as pancreatic ductal carcinoma, Ebola virus infection, Hepatic fibrosis and others [151]. siRNAs as therapeutic options offer the benefit of high target gene specificity and the flexibility to target various aspects of the disease from chronic inflammation to matrix degradation. However, these methods do not come without their shortcomings. siRNAs are negatively charged, making it difficult for them to cross cell membranes and elicit a response. This characteristic causes difficulties in delivery and a lack of adequate volume of siRNA accessing the area of interest. Due to these difficulties, researchers are investigating different drug delivery techniques such as liposome-based, nanoparticle-based and antibody-based methods to provide better localized delivery [148]. Current siRNA targets under investigation in preclinical trials for OA include, but are not limited to, Indian hedgehog, NF-kB, yes-associated protein, hypoxia-induced factor 2 a, and matrix-degrading enzymes such as matrix metalloproteinases [158,160,281,282].

The use of miRNAs is also only in the preclinical stage, with targeting genes such as MMP-13, ADAMTS-5, vascular endothelial growth factor (VEGF), BCL2 and more being used [158,283,284]. lncRNAs have two promising therapeutic uses, as predictive biomarkers, such as HOTTIP, and potential therapies, via the downregulation of lncRNA-CIR, for example^,^ [285,286]. Similarly, circRNAs provide potentials biomarkers, such as hsa_circ 0032131, and as potential therapies [287]. However, research into the use of lnRNAs and circRNAs in this manner is very preliminary and there is a lack of evidence of its efficacy. There is great potential for the use of siRNAs, miRNAs, lncRNAs and circRNAs in the treatment of OA; however, further investigation into the pathogenesis of OA and the use of miRNAs, siRNAs, lncRNAs and circRNAs in treatment is required.

## 10. Concluding Remarks

As the global population ages, the prevalence of age-related diseases, such as OA, is predicted to increase. In the United States alone, the aged population over the age of 85 years is theorized to triple by 2050 [288]. Skeletal and joint diseases such as osteoarthritis reduce mobility in affected individuals due to chronic pain and stiffness, particularly in diarthrodial joints. In this review, we have examined several known epigenetic factors that contribute to OA disease pathogenesis. The dysregulation of these epigenetic regulators contributes to all aspects of OA progression. They regulate proinflammatory cytokine and chemokine expression and secretion, the macrophage inflammatory response within synovial tissues, proteolytic enzymes that increase cartilage degradation, and even the regulation of bone cell homeostasis, which contributes to abnormal subchondral bone remodeling and the formation of osteophytes.

Currently, pharmacological and surgical interventions for OA are limited. They focus primarily on symptom management but are unable to treat the underlying causes of the disease or slow its progression. Pharmacological remedies that address epigenetic regulation offer hope more effective treatments will soon be identified.

## Data Availability

Not applicable.

## Abbreviations

Osteoarthritis (OA), non-coding RNAs (ncRNAs), small-interfering RNAs (siRNAs), microRNAs (miRNAs), extracellular matrix (ECM), polycomb repressive complexes (PRCs), DNA methyltransferases (DNMTs), matrix metalloproteinase (MMP), A disintegrin and metalloproteinase with thrombospondin motif 5 (ADAMTS-5), interleukin (IL), inducible nitric oxide synthase (iNOS), tumor necrosis factor-alpha (TNF-α), type-II collagen (Col2α1), aggrecan (ACAN), sclerostin (SOST), Wnt-ligand secretion mediator (WLS), single nucleotide polymorphisms (SNPs), type-11a1 collagen (COL11a1), vascular endothelial growth factor (VEGF), growth differentiation factor 5 (GDF5), small ubiquitin-like modifiers (SUMO), histone methyltransferases (HMTs), histone demethyltransferases (HDMTs), SRY-box transcription factor-9 (SOX-9), Nuclear Factor of Activated T-cells-1 (NFATC1), DOT1-like histone lysine methyltransferase (DOT1L), sirtuin 1 (SIRT1), lymphoid enhancer-binding factor 1 (LEF1), T-cell factor 1 (TCF1), wingless-type (Wnt), Runt-related transcription factor 2 (RUNX2), Indian hedgehog (IHH), histone acetyltransferases (HATs), histone deacetylases (HDACs), enhancer of Zeste 1 polycomb repressive complex subunit-1 (EZH1), enhancer of Zeste 2 polycomb repressive complex subunit-2 (EZH2), scaffold protein Zeste 12 homolog (SUZ12), nitric oxide (NO), secreted frizzle-related protein (SFRP-1), medial meniscectomy (MMx), conditional knockout (cKO), the hypoxia-inducible transcription factor 1α (Hif1α), small nucleolar RNAs (snoRNAs), small nuclear RNAs (snRNAs), long non-coding RNAs (lncRNAs), circular RNAs (circRNAs), RNA-induced silencing complex (RISC), RNA interference (RNAi), Transforming growth factorβ-activated kinase-1 (TAK1), transforming growth factor-beta (TGF-β), the 3′untranslated region (3′ UTR), insulin like growth factor binding protein 5 (IGFBP-5), BCL2 apoptosis regulator (BCL2), bone morphogenic protein-2 (BMP-2), metastasis-associated lung adenocarcinoma transcript 1 (MALAT1), FOXD2-adjeacent opposite strand RNA 1 (FOXD2-AS1), Toll-like receptor 4 (TRL4), competitive endogenous RNA (ceRNA), ten-eleven-translocation (TET), and mesenchymal stem cells (MSCs).
